# Effect of culling on individual badger *Meles meles* behaviour: Potential implications for bovine tuberculosis transmission

**DOI:** 10.1111/1365-2664.13512

**Published:** 2019-10-08

**Authors:** Cally Ham, Christl A. Donnelly, Kelly L. Astley, Seth Y. B. Jackson, Rosie Woodroffe

**Affiliations:** ^1^ Department of Infectious Disease Epidemiology Science and Solutions for a Changing Planet DTP MRC Centre for Global Infectious Disease Analysis Imperial College London London UK; ^2^ Institute of Zoology Regent's Park, London UK; ^3^ Department of Infectious Disease Epidemiology MRC Centre for Global Infectious Disease Analysis Imperial College London London UK; ^4^ Department of Statistics University of Oxford Oxford UK

**Keywords:** Badger cull, badger *Meles meles*, bovine tuberculosis, perturbation, ranging behaviour, TB, wildlife disease, wildlife management

## Abstract

1. Culling wildlife as a form of disease management can have unexpected and sometimes counterproductive outcomes. In the UK, badgers *Meles meles* are culled in efforts to reduce badger‐to‐cattle transmission of *Mycobacterium bovis*, the causative agent of bovine tuberculosis (TB). However, culling has previously been associated with both increased and decreased incidence of *M. bovis* infection in cattle.
2. The adverse effects of culling have been linked to cull‐induced changes in badger ranging, but such changes are not well‐documented at the individual level. Using GPS‐collars, we characterized individual badger behaviour within an area subjected to widespread industry‐led culling, comparing it with the same area before culling and with three unculled areas.
3. Culling was associated with a 61% increase (95% CI 27%–103%) in monthly home range size, a 39% increase (95% CI 28%–51%) in nightly maximum distance from the sett, and a 17% increase (95% CI 11%–24%) in displacement between successive GPS‐collar locations recorded at 20‐min intervals. Despite travelling further, we found a 91.2 min (95% CI 67.1–115.3 min) reduction in the nightly activity time of individual badgers associated with culling. These changes became apparent while culls were ongoing and persisted after culling ended.
4. Expanded ranging in culled areas was associated with individual badgers visiting 45% (95% CI 15%–80%) more fields each month, suggesting that surviving individuals had the opportunity to contact more cattle. Moreover, surviving badgers showed a 19.9‐fold increase (95% CI 10.8–36.4‐fold increase) in the odds of trespassing into neighbouring group territories, increasing opportunities for intergroup contact.
5. *Synthesis and applications*. Badger culling was associated with behavioural changes among surviving badgers which potentially increased opportunities for both badger‐to‐badger and badger‐to‐cattle transmission of *Mycobacterium bovis*. Furthermore, by reducing the time badgers spent active, culling may have reduced badgers' accessibility to shooters, potentially undermining subsequent population control efforts. Our results specifically illustrate the challenges posed by badger behaviour to cull‐based TB control strategies and furthermore, they highlight the negative impacts culling can have on integrated disease control strategies.

## Graphical abstract

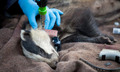

Editor's Choice

## 1 INTRODUCTION

Wildlife populations can transmit infection to economically important domestic species, complicating disease management programmes (Gortazar et al., 2015). For example, wild boar *Sus scrofa* can transmit the Classical Swine Fever virus to domestic pigs (Schulz et al., 2017), and migrating populations of wild birds can transmit avian influenza virus to poultry (Gauthier‐Clerc, Lebarbenchon, & Thomas, 2007).

Where transmission from wildlife to livestock is thought responsible for maintaining infection within livestock, wildlife culling is often attempted (Gortazar et al., 2015; Gortázar, Ferroglio, Höfle, Frölich, & Vicente, 2007). In principle, culling the wildlife species would be expected to reduce transmission from wildlife to livestock both by lowering the infection prevalence within the wildlife species and by reducing opportunities for contact between wildlife and livestock (Barlow, 1996). Wildlife culling has been implemented in attempts to control rabies in red foxes *Vulpes vulpes* (Holmala & Kauhala, 2006), louping ill virus in mountain hares *Lepus timidus* (Harrison, Newey, Gilbert, Haydon, & Thirgood, 2010) and *Mycobacterium bovis* (the causative agent of bovine tuberculosis [TB]) in brushtail possums *Trichosurus vulpecula* (Caley, Hickling, Cowan, & Pfeiffer, 1999). Despite its widespread use, culling has rarely proved successful at reducing the threat of infection from wildlife (Gortazar et al., 2015; Harrison et al., 2010). When wildlife culls fail to contribute to disease management programmes it can be due to both failure in reducing population density and unexpected behavioural and demographic responses of the culled population (Choisy & Rohani, 2006; Streicker et al., 2012). For example, culling red foxes and raccoon dogs *Nyctereutes procyonoides* to control rabies in Europe resulted in increased ranging and dispersal as surviving individuals moved to fill vacated territories, and the re‐establishment of territorial boundaries involved fighting, increasing the risk of rabies transmission (Holmala & Kauhala, 2006).

Bovine tuberculosis (TB) is the most important endemic livestock disease in the United Kingdom. The percentage of cattle herds testing positive for *M. bovis* infection has increased since the 1990s, and 5.8% of herds in England were affected in 2018 (Defra, 2018a). *M. bovis* is a generalist pathogen and has been isolated from a variety of mammal species (Delahay et al., 2007). However, the European badger *Meles meles* has been identified as the predominant wildlife host in Britain (Krebs et al., 1997). It has been estimated that within areas of high TB incidence, 5.7% (95% confidence interval: 0.9%–25%) of transmission into cattle herds is due to badger‐to‐cattle transmission, with cattle‐to‐cattle transmissions amplifying this (Donnelly & Nouvellet, 2013).

Badger culling has been used in Britain and Ireland in attempts to reduce the risk of badger‐to‐cattle transmission of *M. bovis*. However, in England, badger culling has had both positive and negative effects on the incidence of cattle herd breakdowns (Brunton et al., 2017; Donnelly et al., 2003, 2006). Despite the risk that badger culling can increase, as well as decrease, cattle herd breakdowns (Donnelly et al., 2006), current TB control policy in England entails industry‐led widespread badger culling, with over half of the southwest peninsula currently within cull zones (Defra, 2014, 2018b).

In undisturbed populations, badgers form social groups which defend territories through fighting and scent‐marking at communal latrines (Kruuk, 1978; Kruuk & Parish, 1982; Rogers & Cheeseman, 1997). Structuring of the badger population into discrete social groups has important consequences for badger‐to‐badger and badger‐to‐cattle transmission, as it reduces the numbers of both badgers and cattle with which each badger has contact opportunities (Böhm, Hutchings, & White, 2009; Rozins et al., 2018).

The capacity of culling to increase *M. bovis* infection in cattle has been attributed to changes in badger population structure caused by lowering the population density (Woodroffe, Donnelly, Cox, et al., 2006; Woodroffe et al., 2009). In culled areas, badgers' ranging behaviour has been described both by radiotracking individuals, and by mapping the distribution of faecal deposits from social groups fed different coloured baits (Table 1). Although constrained by observation effort and seasonal requirements, both methods have shown that culling is associated with increases in home range size and home range overlap (Carter et al., 2007; O'Corry‐Crowe, Hammond, Eves, & Hayden, 1996; Woodroffe, Donnelly, Cox, et al., 2006). However, it has not been possible to conduct bait‐marking or radiotracking whilst culls were ongoing and therefore behavioural changes occurring during the cull period are unknown (Riordan, Delahay, Cheeseman, Johnson, & Macdonald, 2011; Woodroffe, Donnelly, Cox, et al., 2006). It has been reported that the increase in *M. bovis* prevalence within the badger population is greatest when culls are prolonged (Woodroffe, Donnelly, Jenkins, et al., 2006), suggesting that behavioural change might start during the culling period.

**Table 1:** Summary of previous studies investigating changes in badger ranging behaviour associated with culling. Widespread culling refers to culls aiming to reduce the badger population in areas ≥100 km^2^. Localized culling refers to culls targeting specific TB‐affected farms or farm clusters

Study	Type of culling	Ranging behaviour measure	Individual or social group level	Timescale	Effect
O'Corry‐Crowe et al. (1996)	Widespread	Bait marking	Social group	1 year after culling	33% (95% CI 10%–50%) increase in home range size after first cull
Tuyttens, Delahay, et al. (2000)	Localized	Bait marking	Social group	Between cull seasons for three years	68% increase in home range size after first cull
Woodroffe et al. (2017)	Widespread	Bait marking	Social group	<2 years after cull	180% (95% CI 70%–362%) increase in home range area compared to unculled areas
Woodroffe et al. (2017)	Localized	Bait marking	Social group	<2 years after cull	74% (95% CI 4%–191%) increase home range area compared to unculled areas
Tuyttens, Delahay, et al. (2000)	Localized	Radiotracking	Individual	Three years of culling	Approximately 33% decrease in home range size after two years of culling
Riordan et al. (2011)	Localized	Radiotracking	individual	<1 year after cull	44% increase in summer home range size after cull

Alongside changes in ranging behaviour, culling might be associated with changes in individual nightly activity patterns. Badger populations are largely regulated by food availability (Kruuk & Parish, 1982) and badgers spend a large proportion of their above‐ground time foraging (McClune, Marks, Delahay, Montgomery, & Scantlebury, 2015). As culling reduces the population size, competition for food is likely to decrease, potentially resulting in individuals reducing their activity. Furthermore, ‘bold’ individuals may be more likely to both emerge early and to emerge during any disturbance caused during the culling process and therefore may be more likely to be removed from the population (Tuyttens et al., 1999). As a result, we might expect the nightly activity pattern of the badger population to change in association with culling, which may have important consequences for shooters attempting to locate free‐ranging badgers.

We took advantage of GPS‐collar data collected from badgers living inside a cull zone before, during and after culling was conducted, and from comparable unculled areas, to assess, for the first time, fine‐scale individual changes in badger behaviour during and after culling. We hypothesized that culling would be associated with increased ranging behaviour, that this might commence while culls were ongoing, and that it would increase opportunities for badger‐to‐badger and badger‐to‐cattle contact. We also hypothesized that culling would be associated with reduced nightly activity.

## 2 MATERIALS AND METHODS

### 2.1 Study sites

Data were collected between 2013 and 2017 from four study sites in Cornwall (C2 [5.9 km^2^], C4 [4.7 km^2^], F1 [5.4 km^2^] and F2 [6.5 km^2^]) each consisting of five cattle farms (Table 2). Study sites have been fully described elsewhere (Woodroffe et al., 2016a) but, in summary, each site included both beef and dairy enterprises and the four sites together comprised 10 beef and 10 dairy farms (Table 2). Study sites were located >20 km apart and represented a range of ecological conditions and farming practices.

**Table 2:** Details of the farm types and number of badgers tracked and culled during this study

	Study Site			
	C2	C4	F1	F2
Number of farms				
Beef	2	3	3	2
Dairy	3	2	2	3
Badgers tracked with GPS‐collars				
Pre‐cull period	12	9	15	20
During and post‐cull period	0	7	8	0
GPS‐collared badgers culled under licence 2016–2017				
2016	0	3	0	0
2017	0	2	0	0
Calendar years studied	2013–2015	2014–2017	2013–2017	2013–2015

In 2016, a badger culling licence was granted for North Cornwall, a region which included the C4 study site (Natural England, 2016). Culling took place in September‐October 2016 and September‐October 2017. Following licence requirements (Defra, 2015), badgers were shot either when cage‐trapped or whilst free‐ranging, and carcasses were removed. No culling was licensed at any other study sites (Table 2).

### 2.2 Data collection

Badgers were trapped and handled under licence from the UK Home Office (project licence 70/7482) and Natural England (20,122,772), following ethical review by the Zoological Society of London (projects BPE/0631 and PWE/691). All data were collected with landowner consent.

Badgers were trapped in wire mesh cages and immobilized with an intramuscular injection of medetomidine, ketamine and butorphanol (de Leeuw, Forrester, Spyvee, Brash, & Delahay, 2004). On first capture individuals were fitted with a microchip (FriendChip, Avid PLC) to enable future identification.

A subset of adult badgers was fitted with GPS‐collars (Telemetry Solutions), programmed to record a location every 20‐min between 18:00 hr–06:00 hr GMT following a predetermined schedule, unless an on‐board accelerometer indicated that the badger was inactive. GPS‐collar data were collected throughout the year (Table S1). We removed GPS‐collar locations identified as potentially imprecise using methodology detailed in Woodroffe et al. 2016a, we refer to this process as ‘filtering’ the data. Previous analyses have shown that such filtering did not alter the outcomes of analyses of badger habitat selection or contact with cattle (Woodroffe et al., 2016b, 2017). Badgers were assigned to social groups based on their capture locations and movement data.

We used the GPS‐collar data to derive eight measures of badger behaviour, detailed below, which were the outcome variables for our statistical analyses. We estimated five variables using the filtered GPS‐collar locations; these were (a) monthly home range area, (b) the number of fields visited each month, (c) the maximum distance from the main sett each night, (d) whether the individual trespassed in a neighbouring territory each night and (e) the 20‐min step‐length. We used the unfiltered GPS‐collar data to calculate three measures of badger behaviour unaffected by GPS‐location accuracy; these were (f) nightly emergence time, (g) nightly return time and (h) the duration of activity each night.

We estimated *monthly home ranges* for individual badgers from filtered GPS‐collar data using the Adaptive Local Convex Hull (*a‐LoCoH*) method (Getz et al., 2007). These estimates were generated using the r package tlocoh (Lyons, 2014). Following Woodroffe et al. (2016a, 2016b) we set the *a*‐parameter (the cumulative distance between nearest neighbouring points used to create each hull) at 1,800 m. We used area (km^2^) within the 95% isopleth as the home range area, which was log‐transformed for analysis.

The number of individual *fields visited* each month was counted per individual. We obtained field boundaries from OS maps (Ordnance Survey, 2002) and confirmed the location of each boundary through ground surveys.

We calculated *maximum nightly sett distance* as each badger's maximum distance (m) from its social group main sett. We calculated *20‐min step‐length* as the distance (m) travelled between each pair of consecutive locations recorded 20‐min apart. Both variables were log‐transformed for analysis.

We generated a binary variable describing whether each individual *trespassed* in a neighbouring territory each night. To define territories, we created social group home ranges by combining data from all members of each social group and estimating their combined home range using *a*‐LoCoH as described above. Previous analyses showed that there was little overlap between these group ranges, indicating that they approximate to territories (Woodroffe et al., 2017). We recorded, for each night of tracking, whether an individual badger was recorded as located in any other groups' territory.

For each night, we calculated the *emergence time* of each badger, as the number of minutes after sunset that the first GPS‐collar location was recorded, and the *return time* as the last recorded GPS‐collar location each night (also calculated as minutes after sunset). Finally, we calculated the *activity time* (in minutes) for each badger each night by subtracting the time of the first GPS‐collar location from the time of the last GPS‐collar location recorded.

In addition to these systematically‐recorded measures of movement behaviour, we characterized the change in known location of two badgers in the culled area with failed GPS‐collars, using a combination of trapping and opportunistic camera trap records by citizen scientists. These two badgers did not contribute data to the statistical analyses.

### 2.3 Statistical analyses

We analysed the effects of culling on the eight outcome variables described above using generalized linear mixed‐effects models (GLMMs) fitted using the r package lme4 (Bates, Maechler, Bolker, & Walker, 2014). For the number of fields visited each month, we fitted a GLMM with a Poisson‐error distribution, and for whether a badger trespassed each night we fitted a GLMM with a binomial‐error distribution (logistic regression). For the other six outcome variables, we fitted GLMMs with normally distributed errors.

To explore the effects of culling, we first created a base model for each outcome variable which included site and month as fixed categorical effects, and badger identity as a random effect. Base models did not include a ‘sex’ variable, as including this factor did not significantly improve the fit of any of the models. Additionally, the models of monthly home range area and fields visited per month included the number of nights tracked as a continuous variable, the model of the probability of trespassing included the number of neighbouring social groups as a continuous variable, and the model of 20‐min step‐length included the time of night (in hours, e.g. 01:00, 02:00) as a categorical variable; all were included as fixed effects.

We explored the effects of culling on each measure of behaviour by adding a ‘cull period’ variable to each base model, classifying all observations from September 2016 onwards at site C4 as ‘during and after cull period’ and observations from other sites, and from C4 before September 2016, as ‘no‐cull period’.

The output from the trespassing GLMM (outlined above) was used to estimate the probability of an individual trespassing each night for each month at each site, with and without culling (Table S4).

As we only collected data during and after September 2016 from sites C4 and F1 (Table 2), we performed secondary analyses using only data from these sites, to check the findings of our primary analyses. Firstly, we created a ‘C4 cull‐period’ categorical variable, in which we classified data as ‘outside‐C4‐cull‐period’ if they came from site F1, or from site C4 prior to September 2016, and as ‘inside‐C4‐cull‐period’ if they came from site C4 during and after September 2016. We likewise created a hypothetical ‘F1 cull‐period’ categorical variable, in which we classified data as ‘outside‐F1‐cull‐period’ if they came from site C4, or from site F1 prior to September 2016, and as ‘inside‐F1‐cull‐period’ if they came from site F1 during and after September 2016. We explored the effects of adding these variables to the base models described above.

To investigate whether behavioural changes occurred whilst culling was ongoing, we conducted additional analyses, replacing the two‐level ‘cull period’ variable with a three‐level variable comprising ‘no‐cull’, ‘during‐cull’ and ‘post‐cull’ levels. For this variable we classified all data from sites C2, F1 and F2 and from C4 prior to September 2016 as ‘no‐cull’, data from C4 from the beginning of September to the end of October 2016 as ‘during‐cull’ and data from C4 from the beginning of November 2016 as ‘post‐cull’. This variable was added to the base models for all outcome variables.

## 3 RESULTS

**Figure 1:**
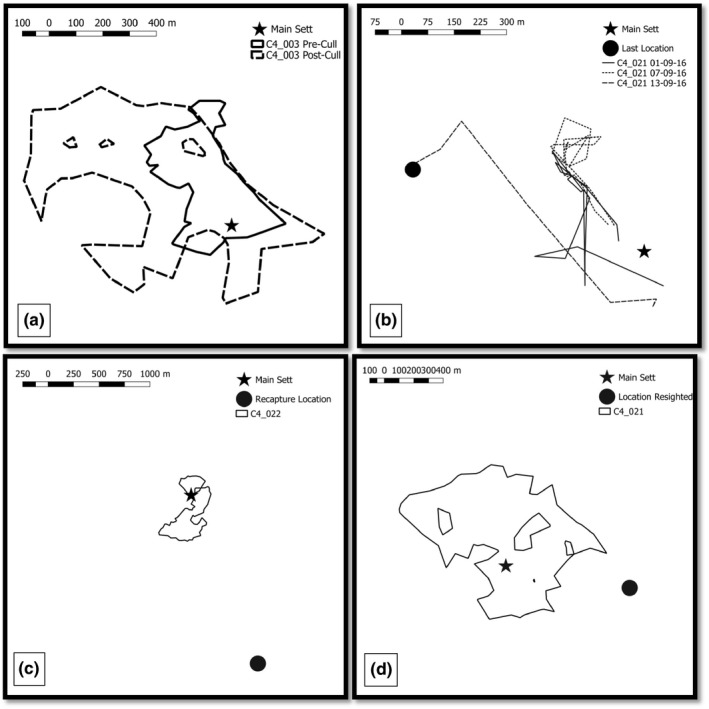
Ranging behaviour of GPS‐collared badgers before, during and after culling at site C4. Panel (a) shows the estimated monthly home range of badger C4_003 in August 2014 (before the cull) and August 2017 (after one year of culling). Both home ranges were constructed using GPS‐collar data collected over 31 nights. Panel (b) shows the nightly tracks of badger C4_021 on: 01‐09‐16 the first night of culling, 07‐09‐16 the seventh night of culling and 13‐09‐16 the night the individual was shot. Panel (c) shows the estimated home range of badger C4_022 between May–August 2016, the location of the main sett where the individual was collared and the location it was recaptured in December 2016. Panel (d) shows the estimated home range of badger C4_023 between May–August 2016, the location of the main sett where the individual was collared and the location it was re‐sighted in April 2017

We collected data over a total of 7,930 badger‐nights during the ‘no‐cull’ period, 69 badger‐nights during the ‘during‐cull’ period and 244 badger‐nights during the ‘post‐cull’ period. This dataset included GPS‐collar information from 67 badgers (Table S1), of which seven were tracked at the culled site (C4) within the ‘during and after cull period’ (Table 2). Figure 1 shows examples of movement behaviour recorded from four of these individuals.

After adjusting for base model variables, culling was significantly associated with increases in all measures of ranging behaviour (Table 3). The ‘during and after cull’ period was associated with a 61% increase (95% CI 27%–103%) in monthly home range area, a 39% (95% CI 28%–51%) increase in nightly maximum distance from the main sett and a 17% (95% CI 11%–24%) increase in 20‐min step‐length. Related to this expanded ranging, we also found a 45% (95% CI 15%–80%) increase in the number of fields visited each month, and a 19.9‐fold increase (95% CI 10.8–36.4‐fold increase) in the odds of trespassing in other group territories. A detailed quantification of how the probability of trespassing varied before and after culling is presented in Table S4.

**Table 3:** Factors associated with badger ranging behaviour. Monthly home range area, 20‐min step‐length and maximum distance from the sett were analysed using linear mixed‐effects regression models, the number of fields visited each month was analysed using a mixed‐effects Poisson regression and individual trespassing was analysed using mixed‐effects logistic regression. Each model also contains badger identity as a random effect

Variable	*df*	Monthly home range area (km^2^)			20‐min step‐length (m)			Nightly maximum distance from sett (m)			Fields visited per month			Probability of trespassing		
		Estimate (95% CI)	*χ* ^2^	*p*	Estimate (95% CI)	*χ* ^2^	*p*	Estimate (95% CI)	*χ* ^2^	*p*	Estimate (95% CI)	*χ* ^2^	*p*	Odds Ratio (95% CI)	*χ* ^2^	*p*
During and After Cull Period	1	61% (27%–103%)	15.13	<0.001	17.0% (11%–24%)	34.17	<0.001	39% (28%–51%)	56.99	<0.001	45% (15%–80%)	9.76	0.002	19.9 (10.8 – 36.4)	121.5	<0.001
Site*	3		5.44	0.14		5.54	0.14		7.75	0.05		25.72	<0.001		25.82	<0.001
Month*	11		102.1	<0.001		1,408	<0.001		507.39	<0.001		82.55	<0.001		219.4	<0.001
Number of Days Tracked (log‐transformed in the analysis of ‘Fields visited per night’)	1	0.01% (0.008–0.02)	25.26	<0.001							25% (21%–28%)	224.9	<0.001			
Hour*	11					96.09	<0.001									
Number of Neighbouring Territories	1													2.2 (1.2–4.1)	6.72	0.01

* Estimates are not presented for site (four‐level categorical variable), month (12‐level categorical variable) or hour of night (12‐level categorical variable).

We also detected evidence of expanded ranging in two badgers with failed GPS‐collars; one was recaptured 1,780 m away from its main sett in December 2016 (Figure 1c), and another was recorded on a camera trap 860m from its pre‐cull home sett in April 2017 (Figure 1d); both records fell outside their pre‐cull home ranges.

The ‘cull period’ variable was also significantly associated with badger emergence time relative to sunset, which was 43.4 min (95% CI 22.7–64.0 min) later during and after culling compared with that in the no‐cull period. The return time relative to sunrise was 51.7 min (95% CI 37.7–65.7 min) earlier during and after culling. Consistent with these patterns, badgers were active for 91.2 min (95% CI 67.1–115.3 min) less during and after culling than in the no‐cull period (Table 4), equivalent to a 22% decrease in individual nightly activity time.

**Table 4:** Factors associated with badger activity. The emergence time, return time and duration of nightly activity were analysed using linear mixed‐effects models. Each model also contains badger identity as a random effect

Variable	*df*	Emergence time (minutes after sunset)			Duration of night activity (minutes)			Return time (minutes after sunset)		
		Estimate (95% CI)	*χ* ^2^	*p*	Estimate (95% CI)	*χ* ^2^	*p*	Estimate (95% CI)	*χ* ^2^	*p*
During and After Cull Period	1	43.4 (22.7–64.0)	16.38	<0.001	−91.2 (−115.29, −67.1)	54.81	<0.001	−51.7 (−65.7, −37.7)	52.10	<0.001
Site*	3		5.71	0.13		0.53	0.91		15.81	0.001
Month*	11		5,980	<0.001		3,195	<0.001		5,828	<0.001

* Estimates are not presented for site (four‐level categorical variable) or month (12‐level categorical variable).

Secondary analyses using only data from sites C4 and F1 confirmed the findings of these primary analyses. As expected, the ‘inside‐C4‐cull‐period’ variable was significantly associated with expanded ranging and reduced activity time (with effect sizes similar to those for the ‘during and after cull period’ variable in the primary analyses), whereas the ‘inside‐F1‐cull‐period’ variable was not (Tables S2 and S3).

Our analyses also revealed that the effects of culling were detectable while culls were ongoing as well as after they were completed. Adding a three‐level cull period variable to the base models revealed that the effects of ‘during‐cull period’ were similar to those for the ‘post‐cull period’ (and to the ‘during and after cull period’ variable in the primary analyses outlined above) for maximum distance each night, trespassing, emergence time, return time and nightly activity. The effect of ‘during‐cull period’ was non‐significant for both the monthly home range area and the number of fields used per month (Table S7).

## 4 DISCUSSION

We found that badger culling was associated with increases in all measures of ranging behaviour among survivors, and reductions in all measures of activity time. These changes became apparent while culls were ongoing and persisted after culling was completed. Our findings of increased ranging associated with culling are consistent with previous studies (Table 1) but provide the first estimates of behavioural change while culling is ongoing. Although our sample of badgers in the culled area was relatively small, confidence in our findings comes from the consistent differences observed, in both primary and secondary analyses, between the behaviour of these animals and the much larger sample of animals not exposed to culling.

The altered ranging behaviour we observed in the culled area potentially increased the intergroup badger contact rate. We found a 19.9‐fold (95% CI 10.8–36.4 fold) increase in the odds of trespassing in the ‘during and after cull period’ compared to the ‘no‐cull period’. The current culling policy aims to reduce the badger population to ≤30% (Defra, 2014); for this to result in an overall decrease in contact between badger social groups (taking into account behavioural change) the increased probability of trespassing in surviving badgers would need to be <3.3, all else being equal. However, we estimated that the increase in the probability (as opposed to the odds) of trespassing at C4 lay in the range 11.9–18.0 (varying between months [Table S4]) suggesting that culling might have prompted an overall increase in badger contact with individuals in neighbouring social groups. In principle, the effect of increased trespassing on the risk of direct badger‐to‐badger transmission might have been reduced by the shorter activity periods associated with culling. However, this argument is undermined by the observation that badgers covered greater distances post‐cull, albeit in less time. Likewise, reduced activity time is unlikely to have reduced opportunities for indirect transmission among badgers, because the behaviours likely to contaminate the environment (foraging, defecation, urination) occur outside the sett and presumably were concentrated into a shorter activity time, albeit over a wider area. Overall, these observed changes in intergroup contact opportunities may help to explain the increase in *M. bovis* prevalence recorded within the badger population following culling (Woodroffe et al., 2009; Woodroffe, Donnelly, Jenkins, et al., 2006).

The altered ranging behaviour also had potential consequences for badger‐to‐cattle contact. Within the culled area, individual badgers visited 45% (95% CI 15%–80%) more fields per month than did individuals living in unculled areas. Assuming cattle use of fields is unchanged by badger culling, this behavioural change provides the opportunity for each badger to contact a greater number of grazing cattle. If the badger population was reduced to 30% of its original size (the aim of the current policy), each badger would need to visit 233% more fields to maintain the same level of badger‐to‐cattle contact opportunity as occurred at the original population size. However, if the badger population were reduced to 75% of the original population (as under localized culling in the Randomized Badger Culling Trial (RBCT; Woodroffe et al., 2008)), individual badgers would only need to visit 33% more fields to maintain the badger‐to‐cattle contact rate, assuming the behavioural change was the same. These calculations suggest that, under localized culling, the 45% increase in field use that we observed might potentially increase opportunities for badger‐cattle contact (although the 33% threshold fell within the 95% confidence interval around the estimate of 45%). In the RBCT, localized culling was associated with a 27% (95% CI 4.8%–53%) increase in cattle herd breakdowns (Donnelly et al., 2003). This increased cattle TB incidence could be at least partially explained by the increased ranging that we describe. However, increased ranging and trespassing are likely to signal a breakdown in territorial behaviour, a change which has been linked to an increase in badger defecations and urinations away from territorial latrines, increasing cattle exposure to potentially infectious excreta (Hutchings, Service, & Harris, 2002). Whilst the same behavioural changes would occur under widespread culling, the population reduction may be sufficient to outweigh cull‐induced increases in badger‐to‐cattle contact. Individual badgers' expanded use of fields in culled areas also provides the opportunity for greater cattle‐to‐badger transmission of *M. bovis*, which may contribute to the recorded increase in infection prevalence among surviving badgers (Woodroffe, Donnelly, Jenkins, et al., 2006).

Our finding that badger behaviour changed while culls were ongoing may have important implications for TB control. Even if an individual is culled during the latter stages of a cull (which under current policy last ≤6 weeks), the behavioural changes exhibited by the individual prior to death may mean that even individuals which are ultimately killed may contribute to increased transmission risk while culls are ongoing. Furthermore, as badger‐to‐cattle transmission is most likely to occur through indirect contact (Woodroffe et al., 2016a) and *M. bovis* can remain viable within the environment for extended periods of time (King et al., 2015), the effects of expanded ranging behaviour could create a source of infection to cattle for several months, even after an individual was culled. The importance of this effect depends on the speed at which the badger population is reduced during the cull. Evidence suggests that the current culls reduce the badger population at a slower rate than the experimental culls which they were intended to emulate (AHVLA, 2015a, 2015b, 2015c; Woodroffe et al., 2008), providing a greater period of time for badgers exhibiting increased ranging behaviour to come into contact with an increased number of badgers and cattle.

Although surviving badgers travelled further within the ‘during and after cull’ period, they were active for less time each night. This reduction in nightly activity might be due to culling selectively removing ‘bold’ individuals that emerge earlier or are active for longer and would be at greatest risk from shooters and traps. Alternatively, individuals living within culled areas might be active for less time each night because they have greater access to food either due to having a larger area over which to forage and/or reduced intraspecific competition within their original home range. Previously it has been found that surviving badgers within a culled area are heavier and in better body condition, suggesting that food availability is altered by culling (Tuyttens, MacDonald, Rogers, Cheeseman, & Roddam, 2000). Whatever the reason, reductions in badgers' active periods is likely to reduce their exposure to shooters, potentially reducing the proportion killed on subsequent culls.

In general a greater proportion of culled badgers were removed by free‐shooting as opposed to cage‐trapping after the first year of culling (Table S5), assuming equal trapping effort; this may reflect the badger population becoming increasingly trap‐shy after culling (Tuyttens et al., 1999). Locating free‐ranging badgers that travel further, more quickly and for a shorter time period each night may make it more difficult to maintain population reduction over successive annual culls.

Our results help to explain why cull‐induced changes to badger behaviour might offset benefits that reducing the overall badger population has towards lowering the incidence of TB within cattle. In contrast to the behavioural changes described in this study, badger vaccination has been shown to generate no such changes in individual ranging behaviour (Woodroffe et al., 2017). A comparison of the movements of badgers in culled and vaccinated populations, updated from Woodroffe et al., 2017, is provided in Table S6.

The results described here highlight how understanding individual behavioural responses to management programmes can be important for understanding the outcomes of such programmes. Understanding the opportunities for changes in contact rates before, during and after a culling programme could provide an insight into how disease transmission might change, especially for group‐living species. An understanding of the timescale over which behavioural changes might occur is important for designing culling protocols to be used. As GPS‐technology becomes more widely available gathering the data needed to understand fine‐scale individual behaviour will become easier and wildlife management protocols should take advantage of this wherever possible.

## AUTHORS' CONTRIBUTIONS

C.H., C.A.D. and R.W. designed the study, C.H., K.L.A., S.Y.B.J. and R.W. collected data, C.H. conducted data analyses and wrote the manuscript with guidance from C.A.D. and R.W. All authors gave final approval for publication.

## Data Availability

All measures of movement behaviour are available on Dryad Digital Repository https://doi.org/10.5061/dryad.1b58p8m (Ham, Donnelly, Astley, Jackson, & Woodroffe, 2019). Raw GPS‐collar data are available on Movebank project 158275131 subject to a confidentiality agreement due to sensitive nature of the data.
