# Supplementary material for: Effect of culling on individual badger Meles meles behaviour: Potential implications for bovine tuberculosis transmission
Source: J Appl Ecol. 2019 Oct 8;56(11):2390–9. doi: 10.1111/1365-2664.13512 (PMC8447922; doi:10.1111/1365-2664.13512)
Supplement: Supplementary file 1 [file JPE-56-2390-s001.docx]

**Effect of Culling on Individual Badger (*Meles meles*) Behaviour: Potential Implications for Bovine Tuberculosis Transmission**

Cally Ham, Christl A Donnelly, Kelly L Astley, Seth YB Jackson, Rosie Woodroffe

**Supporting Information**

*Contents*

1. Additional analyses
   1. Comparison of monthly home range area in control, culled and vaccinated areas
   2. Comparison of individual ranging behaviour and nightly activity pattern in “pre-cull”, “during-cull” and “post-cull” periods
2. Additional tables
3. Supporting information references

*1a Comparison of monthly home range area in control, culled and vaccinated areas*

To compare the effects of badger vaccination and badger culling on individual monthly home range area we created a “treatment” variable and assigned individual badgers to a treatment based on the management conducted at their capture location. Individuals from:

Site C2

Sites F1 and F2 prior to September 2014

Site C4 prior to September 2016

were classified as “control” as the site had not been subject to either culling or vaccination. Individuals from sites F1 and F2 after September 2014 were classified as “vaccination”. Individuals from site C4 after September 2016 were classified as “culled”.

We used this “treatment” variable to test for an effect of vaccination or culling on individual monthly home range area, using home ranges created using *a*-LoCoH (Getz *et al.* 2007) as described in the main text, using a generalized linear mixed model (GLMM) fitted with a normal error distribution. The model also contained fixed effects of “site” and “month” as categorical variables and “number of days tracked” as a numeric variable. We also included badger identity as a random effect.

We found that treatment had a significant effect on individual monthly home range area (X^2^=14.18, DF=2, P<0.001) (Table S6).

*1b Comparison of individual badger behaviour within the “pre-cull”, “during cull” and “post-cull” periods*

To distinguish between individual behavioural changes occurring whilst culling was ongoing compared to behavioural changes occurring between cull periods we created a new “cull period” variable. We classified all data collected from sites C2, F1 and F2 and data collected from site C4 before September 2016 as within the “no-cull” period. We classified data collected from C4 during September and October 2016 as from within the “during-cull” period. Finally, we classified data from C4 between November 2016 and September 2017 as “post-cull” period.

We tested the effect of the “cull period” variable on all measures of badger behaviour. We used logistic regression to analyse trespassing, GLMM fitted with Poisson error structure for the variable of number of fields visited per month and GLMMs fitted with normally distributed errors for the remaining six variables of; 20-minute step length, nightly maximum distance from the main sett, emergence time, nightly activity time and return time. In each case we included all base model variables described in the main text and badger identity as a random effect. We found a significant difference between the pre-cull, during-cull and post-cull periods on each measure of badger behaviour except for the monthly home range area, number of fields visited per month and the 20-minute step-length for which we found no significant effect of the during-cull period (Table S7).

*2: Additional Tables*

Table S1: The data collection period(s) for each individual badger recorded in this study alongside the number of GPS-collar locations recorded for both the “filtered” and “unfiltered” data sets. Underlining indicates individual shot during the cull and the collar period during which it was shot.

| ID | **Sex** | **Start Date** | **End Date** | **Days Tracked** | **Number locations (filtered)** | **Number locations (unfiltered)** |
| --- | --- | --- | --- | --- | --- | --- |
| C2_002 | Male | 23/05/2013 | 01/08/2013 | 70 | 651 | 886 |
| C2_003 | Male | 22/05/2013 | 24/09/2013 | 125 | 1592 | 2038 |
| C2_004 | Female | 22/05/2013 | 01/07/2013 | 40 | 477 | 559 |
| C2_005 | Male | 23/05/2013 | 15/09/2013 | 115 | 1189 | 1499 |
| C2_006 | Male | 23/05/2013 | 04/07/2013 | 42 | 294 | 333 |
| C2_008 | Female | 24/05/2013 | 02/09/2013 | 101 | 1401 | 1837 |
| C2_011 | Male | 11/01/2014 | 23/03/2014 | 71 | 766 | 935 |
| C2_011 | Male | 05/06/2014 | 05/10/2014 | 122 | 1758 | 2304 |
| C2_015 | Male | 10/01/2014 | 17/04/2014 | 97 | 813 | 979 |
| C2_015 | Male | 09/06/2014 | 09/10/2014 | 122 | 2233 | 2840 |
| C2_017 | Male | 11/01/2014 | 09/06/2014 | 149 | 1870 | 2413 |
| C2_017 | Male | 09/06/2014 | 24/10/2014 | 137 | 2586 | 3213 |
| C2_019 | Male | 23/01/2014 | 24/04/2014 | 91 | 1020 | 1380 |
| C2_020 | Female | 05/06/2014 | 12/06/2014 | 7 | 70 | 94 |
| C2_022 | Male | 23/01/2015 | 11/05/2015 | 108 | 1547 | 1971 |
| C4_001 | Female | 14/07/2014 | 04/09/2014 | 52 | 796 | 1001 |
| C4_003 | Female | 17/07/2014 | 16/12/2014 | 152 | 3260 | 3693 |
| C4_003 | Female | 17/06/2017 | 22/09/2017 | 97 | 1690 | 1970 |
| C4_004 | Female | 17/07/2014 | 12/09/2014 | 57 | 883 | 1180 |
| C4_004 | Female | 30/09/2014 | 03/02/2015 | 126 | 955 | 1169 |
| C4_004 | Female | 24/05/2016 | 22/08/2016 | 90 | 1172 | 1388 |
| C4_004 | Female | 23/08/2016 | 27/09/2016 | 35 | 601 | 687 |
| C4_005 | Female | 30/09/2014 | 25/10/2014 | 25 | 405 | 500 |
| C4_006 | Male | 02/10/2014 | 18/12/2014 | 77 | 1510 | 1724 |
| C4_007 | Female | 17/06/2017 | 08/07/2017 | 21 | 251 | 315 |
| C4_008 | Female | 01/10/2014 | 23/10/2014 | 22 | 461 | 550 |
| C4_013 | Female | 23/08/2016 | 21/12/2016 | 120 | 372 | 453 |
| C4_021 | Female | 23/05/2016 | 24/08/2016 | 93 | 1390 | 1918 |
| C4_021 | Female | 24/08/2016 | 14/09/2016 | 21 | 426 | 483 |
| C4_022 | Female | 23/05/2016 | 24/08/2016 | 93 | 1645 | 1250 |
| C4_023 | Male | 23/05/2016 | 24/08/2016 | 93 | 989 | 181 |
| C4_024 | Male | 24/08/2016 | 03/09/2016 | 10 | 144 | 181 |
| C4_025 | Male | 17/06/2017 | 19/09/2017 | 94 | 1334 | 1609 |
| F1_002 | Male | 14/05/2013 | 08/11/2013 | 178 | 2605 | 3032 |
| F1_003 | Male | 14/05/2013 | 14/09/2013 | 123 | 1759 | 2063 |
| F1_003 | Male | 22/09/2014 | 10/05/2015 | 230 | 3089 | 3465 |
| F1_004 | Male | 13/05/2013 | 06/06/2013 | 24 | 168 | 211 |
| F1_006 | Female | 16/05/2013 | 06/08/2013 | 82 | 1189 | 1410 |
| F1_006 | Female | 13/11/2013 | 14/02/2014 | 93 | 856 | 987 |
| F1_006 | Female | 22/09/2014 | 25/02/2015 | 156 | 2094 | 2420 |
| F1_009 | Female | 15/07/2017 | 05/09/2017 | 52 | 1052 | 1178 |
| F1_010 | Female | 17/06/2014 | 17/06/2014 | 0 | 5 | 12 |
| F1_013 | Female | 17/05/2013 | 21/08/2013 | 96 | 1274 | 1585 |
| F1_015 | Female | 17/05/2013 | 06/09/2014 | 477 | 2779 | 3373 |
| F1_015 | Female | 29/10/2013 | 12/03/2014 | 134 | 1330 | 1605 |
| F1_020 | Female | 16/06/2014 | 18/11/2014 | 155 | 3313 | 3890 |
| F1_022 | Male | 27/10/2013 | 31/01/2014 | 96 | 1294 | 1581 |
| F1_024 | Female | 26/11/2014 | 03/03/2015 | 97 | 966 | 1111 |
| F1_029 | Male | 22/09/2014 | 07/02/2015 | 138 | 1942 | 2252 |
| F1_030 | Female | 24/09/2014 | 18/12/2014 | 85 | 1328 | 1561 |
| F1_033 | Female | 16/06/2014 | 08/04/2015 | 296 | 3897 | 4420 |
| F1_036 | Male | 22/09/2014 | 09/11/2014 | 48 | 976 | 1136 |
| F1_037 | Male | 15/07/2017 | 05/09/2017 | 52 | 641 | 508 |
| F1_039 | Female | 18/06/2014 | 24/10/2014 | 128 | 2847 | 3242 |
| F1_039 | Female | 04/10/2016 | 15/07/2017 | 284 | 4873 | 5521 |
| F1_046 | Male | 04/10/2016 | 24/11/2016 | 51 | 1011 | 1167 |
| F1_064 | Male | 05/10/2016 | 09/02/2017 | 127 | 2544 | 844 |
| F1_065 | Female | 15/07/2017 | 05/09/2017 | 52 | 954 | 265 |
| F1_066 | Female | 04/10/2016 | 24/02/2017 | 143 | 1873 | 1049 |
| F1_070 | Female | 15/07/2017 | 05/09/2017 | 52 | 792 | 179 |
| F2_002 | Male | 10/09/2013 | 16/11/2013 | 67 | 498 | 632 |
| F2_004 | Male | 10/09/2013 | 24/02/2014 | 167 | 2179 | 2694 |
| F2_005 | Male | 10/09/2013 | 22/01/2014 | 134 | 1741 | 2245 |
| F2_007 | Female | 10/09/2013 | 14/05/2014 | 246 | 3124 | 3738 |
| F2_012 | Female | 16/09/2013 | 30/10/2013 | 44 | 807 | 953 |
| F2_015 | Male | 19/09/2013 | 12/03/2014 | 174 | 2220 | 2857 |
| F2_017 | Male | 25/09/2013 | 30/10/2013 | 35 | 406 | 480 |
| F2_020 | Female | 07/05/2014 | 09/09/2014 | 125 | 1989 | 2546 |
| F2_023 | Female | 10/09/2014 | 27/01/2015 | 139 | 1243 | 1436 |
| F2_023 | Female | 28/01/2015 | 08/06/2015 | 131 | 1839 | 2248 |
| F2_024 | Male | 07/05/2014 | 21/01/2015 | 259 | 3405 | 4259 |
| F2_025 | Female | 08/05/2014 | 10/09/2014 | 125 | 1831 | 2366 |
| F2_025 | Female | 10/09/2014 | 27/01/2015 | 139 | 1578 | 1856 |
| F2_026 | Female | 07/05/2014 | 14/05/2014 | 7 | 59 | 92 |
| F2_030 | Female | 13/05/2014 | 27/05/2014 | 14 | 166 | 213 |
| F2_032 | Female | 14/05/2014 | 18/05/2014 | 4 | 38 | 49 |
| F2_032 | Female | 30/06/2014 | 24/07/2014 | 24 | 326 | 425 |
| F2_032 | Female | 08/09/2014 | 21/10/2014 | 43 | 1057 | 1229 |
| F2_033 | Female | 14/05/2014 | 03/07/2014 | 50 | 821 | 1065 |
| F2_034 | Female | 15/05/2014 | 12/06/2014 | 28 | 360 | 485 |
| F2_034 | Female | 08/09/2014 | 27/04/2015 | 231 | 3010 | 3408 |
| F2_039 | Male | 01/07/2014 | 27/01/2015 | 210 | 3441 | 4121 |
| F2_041 | Female | 09/09/2014 | 18/12/2014 | 100 | 811 | 1044 |
| F2_043 | Male | 24/01/2015 | 17/03/2015 | 52 | 724 | 940 |
| F2_045 | Female | 28/01/2015 | 11/06/2015 | 134 | 1424 | 1994 |

Table S2: Factors associated with badger ranging behaviour in the “C4 cull period” and hypothetical “F1 cull period”. Monthly home range area, 20-minute step-length and maximum distance from the sett were analysed using GLMM, the number of fields visited per month was analysed using a mixed effects Poisson regression and individual trespassing was analysed using mixed-effects logistic regression. Each model contains the same fixed and random effects as those presented in Table 3 and uses data from sites C4 and F1 only.

| Variable | D.F. | Monthly Home Range Area (km^2^) | | | Step-Length (m) | | | Maximum Distance From Sett (m) | | | Fields Visited Per Month | | | Probability of Trespassing | | |
| --- | --- | --- | --- | --- | --- | --- | --- | --- | --- | --- | --- | --- | --- | --- | --- | --- |
|  |  | Estimate (95%CI) | X^2^ | P Value | Estimate (95%CI) | X^2^ | P Value | Estimate (95%CI) | X^2^ | P Value | Estimate (95%CI) | X^2^ | P Value | Odds Ratio (95%CI) | X^2^ | P Value |
| C4 Cull Period | 1 | 61% (24-110%) | 13.33 | <0.001 | 17% (11-23%) | 35.06 | <0.001 | 38% (25-51%) | 45.59 | <0.001 | 42% (13-79%) | 8.87 | 0.003 | 23.02 (12.4 – 42.9) | 129.72 | <0.001 |
| F1 Cull Period | 1 | -25% (-43 - -0.01%) | 4.02 | 0.04 | 4% (-0.01 – 10%) | 2.42 | 0.12 | 0.05% (-0.09 – 11%) | 0.002 | 0.97 | -10% (-24 – 6%) | 1.55 | 0.21 | 1.1 (0.6 – 1.8) | 0.06 | 0.81 |

Table S3: Factors associated with badger activity patterns in the “C4 cull period” and hypothetical “F1 cull period”. The emergence time, return time and duration of nightly activity were analysed using GLMMs. Each model contains the same fixed and random effects as those presented in Table 4 and uses data from sites C4 and F1 only.

| Variable | D.F. | Emergence Time (minutes after sunset) | | | Night Duration (minutes) | | | Return Time (Minutes after sunset) | | |
| --- | --- | --- | --- | --- | --- | --- | --- | --- | --- | --- |
|  |  | Estimate (95%CI) | X^2^ | P Value | Estimate (95%CI) | X^2^ | P Value | Estimate (95%CI) | X^2^ | P Value |
| C4 Cull Period | 1 | 36.27 (15.98-56.56) | 12.22 | <0.001 | -89.63 (-113.83 - -65.43) | 52.59 | <0.001 | -57.90 (-73.13 - -42.66) | 55.29 | <0.001 |
| F1 Cull Period | 1 | -22.32 (-43.07- -1.58) | 4.28 | 0.04 | 6.02 (-19.22 – 31.26) | 0.18 | 0.67 | -17.51 (-32.91 - -2.10) | 5.04 | 0.02 |

Table S4: The estimated probabilities for individual trespassing at each site before and after culling, calculated for every month at each of the four study sites.

| Site | Month | Probability of trespassing pre-cull | Probability of trespassing during and post-cull | Ratio of probabilities |
| --- | --- | --- | --- | --- |
| C2 | 1 | 0.03 | 0.40 | 12.27 |
|  | 2 | 0.08 | 0.62 | 8.08 |
|  | 3 | 0.07 | 0.58 | 8.85 |
|  | 4 | 0.07 | 0.58 | 8.87 |
|  | 5 | 0.05 | 0.49 | 10.64 |
|  | 6 | 0.03 | 0.42 | 12.01 |
|  | 7 | 0.07 | 0.58 | 8.90 |
|  | 8 | 0.10 | 0.70 | 6.72 |
|  | 9 | 0.14 | 0.76 | 5.54 |
|  | 10 | 0.07 | 0.59 | 8.74 |
|  | 11 | 0.05 | 0.52 | 9.96 |
|  | 12 | 0.02 | 0.32 | 13.79 |
| C4 | 1 | 0.01 | 0.13 | 17.33 |
|  | 2 | 0.02 | 0.28 | 14.64 |
|  | 3 | 0.02 | 0.24 | 15.26 |
|  | 4 | 0.02 | 0.24 | 15.28 |
|  | 5 | 0.01 | 0.18 | 16.46 |
|  | 6 | 0.01 | 0.14 | 17.20 |
|  | 7 | 0.02 | 0.24 | 15.30 |
|  | 8 | 0.03 | 0.35 | 13.34 |
|  | 9 | 0.04 | 0.42 | 11.94 |
|  | 10 | 0.02 | 0.25 | 15.18 |
|  | 11 | 0.01 | 0.20 | 16.04 |
|  | 12 | 0.01 | 0.10 | 18.00 |
| F1 | 1 | 0.18 | 0.81 | 4.58 |
|  | 2 | 0.35 | 0.91 | 2.63 |
|  | 3 | 0.31 | 0.90 | 2.91 |
|  | 4 | 0.31 | 0.90 | 2.92 |
|  | 5 | 0.23 | 0.86 | 3.67 |
|  | 6 | 0.18 | 0.82 | 4.42 |
|  | 7 | 0.31 | 0.90 | 2.93 |
|  | 8 | 0.42 | 0.94 | 2.21 |
|  | 9 | 0.50 | 0.95 | 1.90 |
|  | 10 | 0.31 | 0.90 | 2.87 |
|  | 11 | 0.26 | 0.87 | 3.36 |
|  | 12 | 0.13 | 0.75 | 5.72 |
| F2 | 1 | 0.17 | 0.81 | 4.66 |
|  | 2 | 0.34 | 0.91 | 2.67 |
|  | 3 | 0.30 | 0.90 | 2.96 |
|  | 4 | 0.30 | 0.90 | 2.96 |
|  | 5 | 0.23 | 0.86 | 3.73 |
|  | 6 | 0.18 | 0.81 | 4.50 |
|  | 7 | 0.30 | 0.90 | 2.97 |
|  | 8 | 0.42 | 0.93 | 2.24 |
|  | 9 | 0.49 | 0.95 | 1.92 |
|  | 10 | 0.31 | 0.90 | 2.91 |
|  | 11 | 0.26 | 0.87 | 3.42 |
|  | 12 | 0.13 | 0.75 | 5.81 |

Table S5: The number of badgers culled through cage-trapping and free-shooting in Cornwall in 2016 and 2017. Numbers published by Defra (Defra 2016, 2017).

| Year | Number removed by cage-trapping | Number removed by free-shooting | Total number removed |
| --- | --- | --- | --- |
| 2016 | 1051 | 510 | 1561 |
| 2017 | 199 | 372 | 571 |

Table S6: The estimated effects of badger culling or badger vaccination tested using a reduced mixed-effects model with fixed effects for treatment, site, month and the number of days tracked and badger identity as a random effect. *Estimates are not presented for site (four-level categorical variable) or month (12-level categorical variable).

| Variable | | D.F. | Estimate | X^2^ | P Value |
| --- | --- | --- | --- | --- | --- |
| Treatment | Culled vs Control | 2 | 63% (26-212%) | 14.18 | <0.001 |
|  | Vaccinated vs Control |  | -0.01% (-14 – 14%) |  |  |
| Site* |  | 3 |  | 5.47 | 0.14 |
| Month* |  | 11 |  | 94.45 | <0.001 |
| Days Tracked |  | 1 | 0.01% (0.009-0.02%) | 30.44 | <0.001 |

Table S7: The estimated effects of “cull period” (no-cull, during-cull or post-cull) on eight measures of badger behaviour analysed using GLMMs. Each model contains the base-model variables outlined in the main text.

| Variable | Treatment | Estimate (95% CI) | P value | t statistic | X^2^ | D.F. |
| --- | --- | --- | --- | --- | --- | --- |
| 20-minute step-length (m) | During cull vs pre-cull | 6% (-3-16%) | 0.18 | 1.35 | 35.58 | 2 |
|  | Post-cull vs pre-cull | 24% (16-33%) | <0.001 | 6.33 |  |  |
| Nightly maximum distance from sett (m) | During cull vs pre-cull | 32% (16-50%) | <0.001 | 4.21 | 57.93 | 2 |
|  | Post-cull vs pre-cull | 44% (29-60%) | <0.001 | 6.64 |  |  |
| Probability of Trespassing (odds ratio) | During cull vs pre-cull | odds ratio  9.9 (4.3 – 22.8) | <0.001 | 5.36 | 129.77 | 2 |
|  | Post-cull vs pre-cull | odds ratio  45.2 (17.6 – 116.3) | <0.001 | 7.90 |  |  |
| Emergence time (minutes after sunset) | During cull vs pre-cull | 46.5 (14.3-78.7) | 0.004 | 2.83 | 16.92 | 2 |
|  | Post-cull vs pre-cull | 41.5 (16.6-66.4) | 0.001 | 3.27 |  |  |
| Duration of night activity (minutes) | During cull vs pre-cull | -75.2 (-113.0 - -37.4) | <0.001 | -3.90 | 55.91 | 2 |
|  | Post-cull vs pre-cull | -99.8 (-128.6 - -71.0) | <0.001 | -6.79 |  |  |
| Return time (minutes after sunset) | During cull vs pre-cull | -34.2 (-56.1 -12.3) | 0.002 | -3.06 | 56.13 | 2 |
|  | Post-cull vs pre-cull | -61.7 (-78.6 - -44.8) | <0.001 | -7.17 |  |  |
| Monthly home range area (km^2^) | During cull vs pre-cull | 14% (-20 – 62%) | 0.48 | 0.70 | 23.06 | 2 |
|  | Post-cull vs pre-cull | 135% (65 – 234%) | <0.001 | 4.76 |  |  |
| Number of fields visited per month | During cull vs pre-cull | 34% (-4 – 86%) | 0.09 | 1.72 | 10.16 | 2 |
|  | Post-cull vs pre-cull | 52% (15 – 102%) | 0.004 | 2.90 |  |  |
